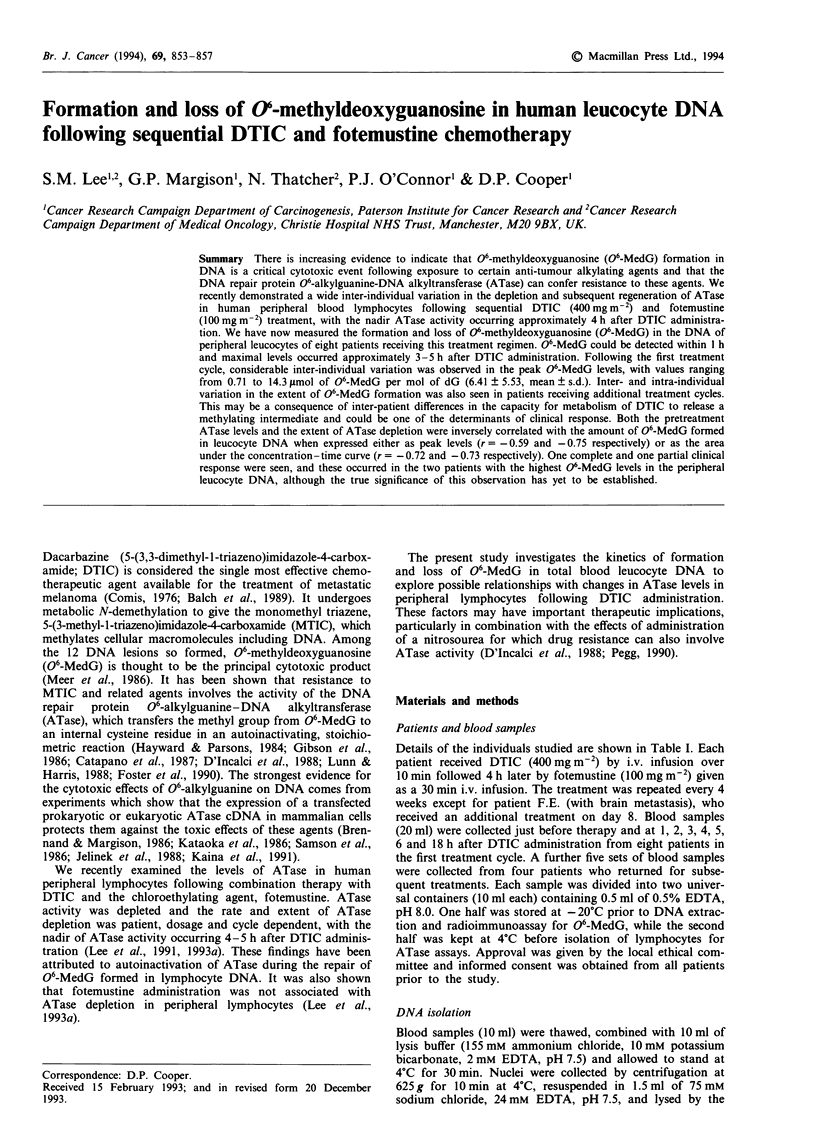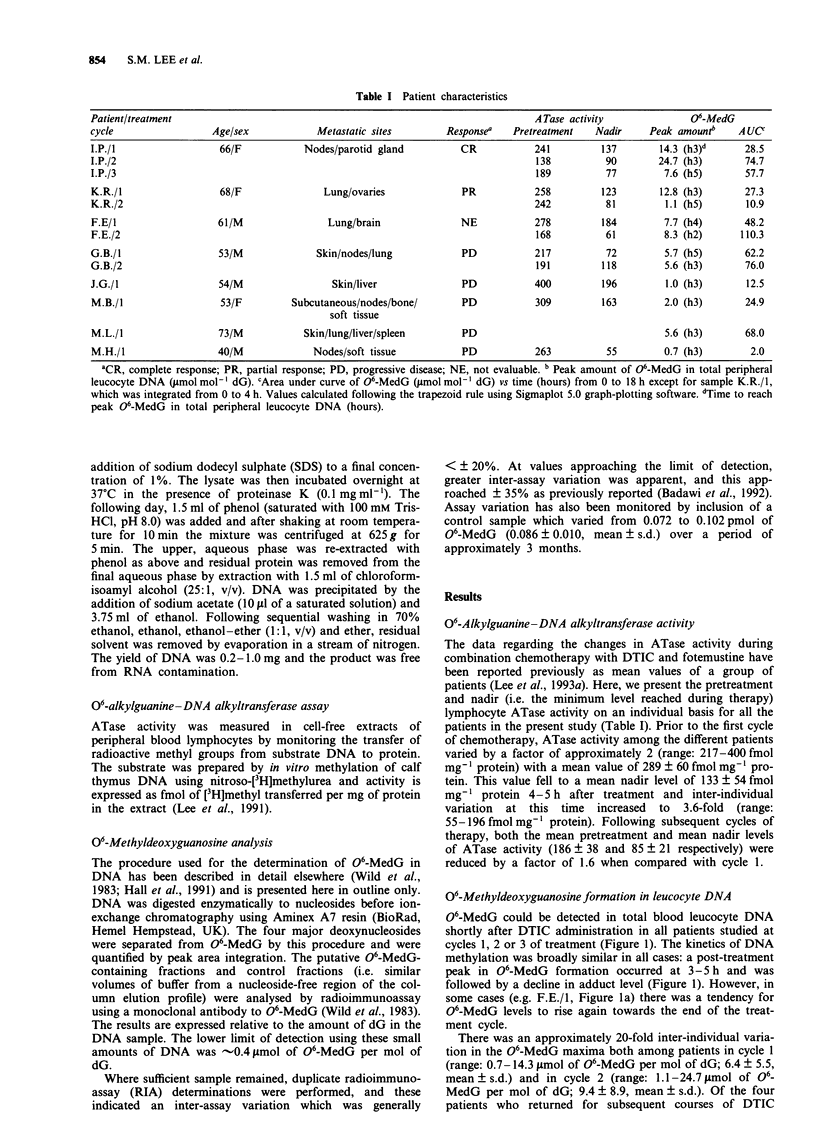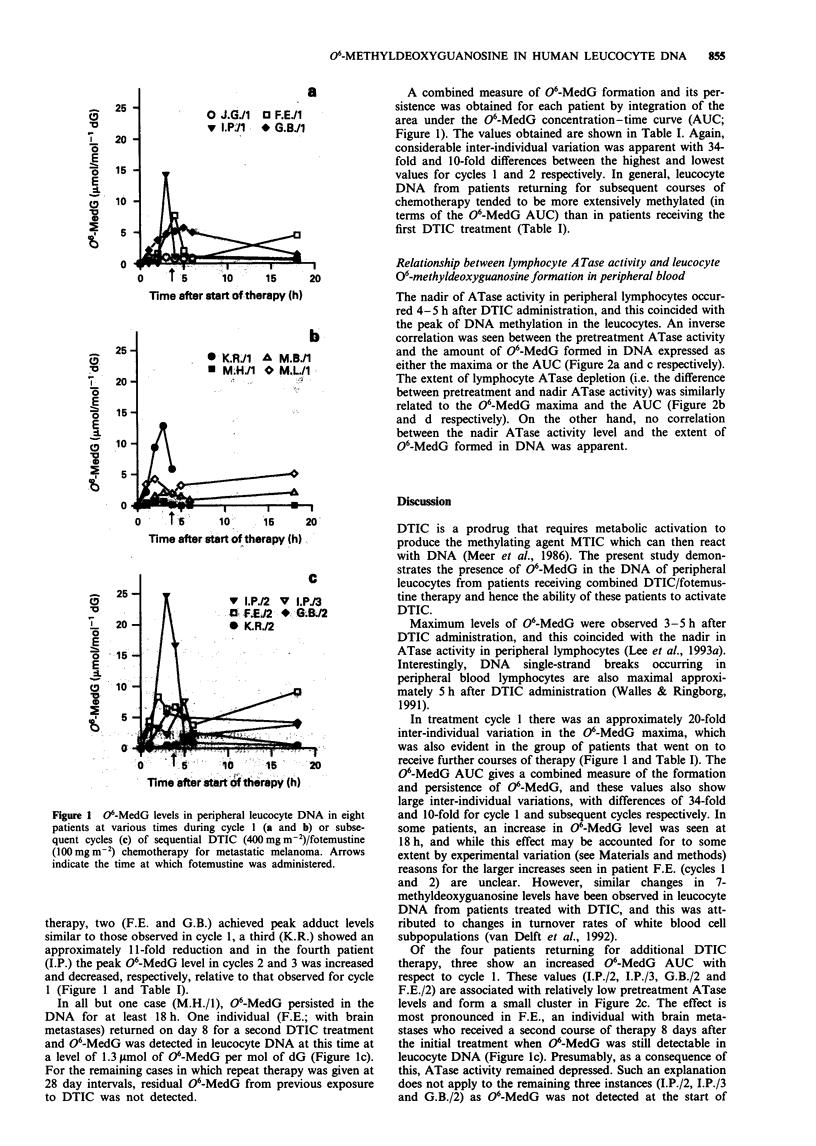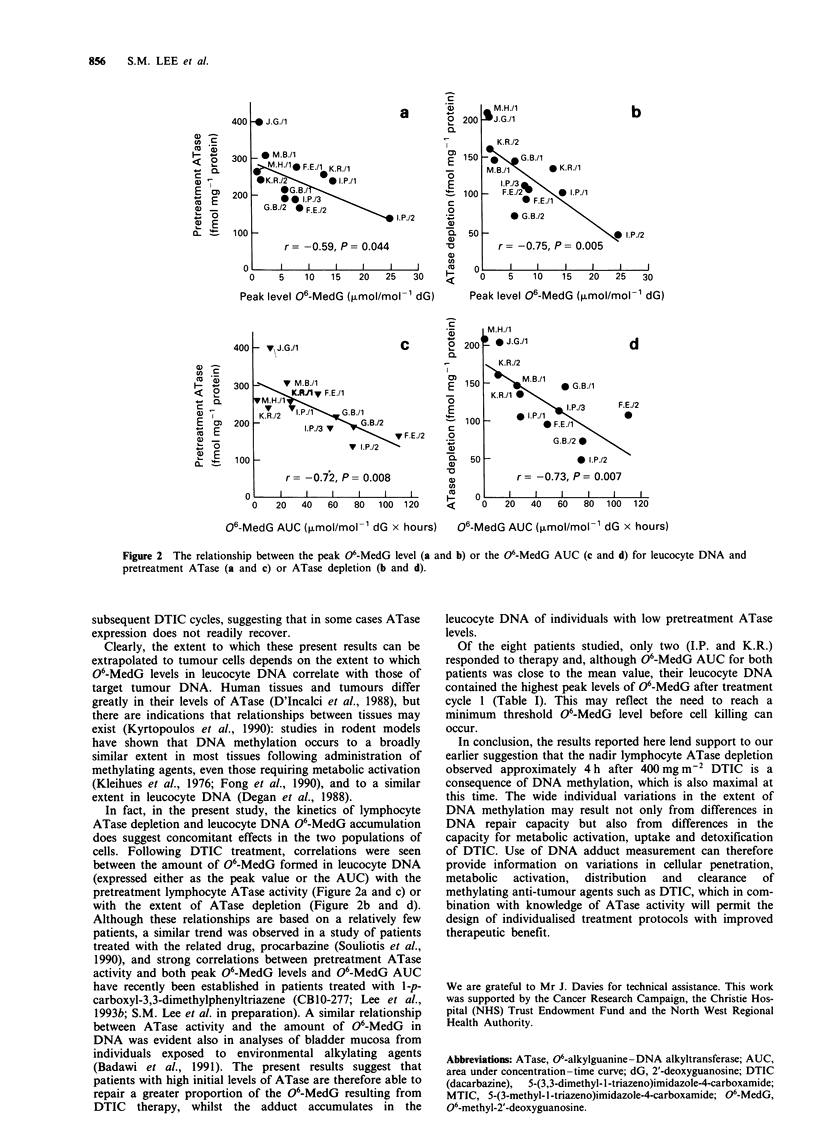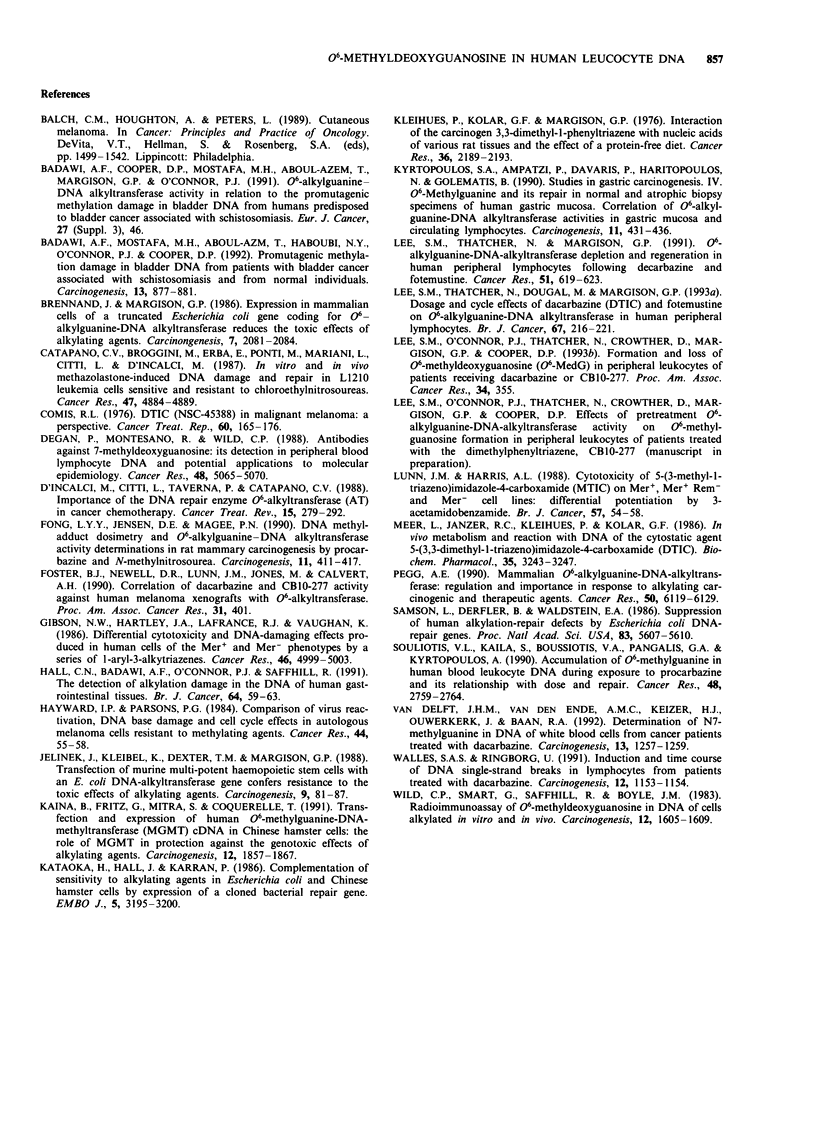# Formation and loss of O6-methyldeoxyguanosine in human leucocyte DNA following sequential DTIC and fotemustine chemotherapy.

**DOI:** 10.1038/bjc.1994.165

**Published:** 1994-05

**Authors:** S. M. Lee, G. P. Margison, N. Thatcher, P. J. O'Connor, D. P. Cooper

**Affiliations:** Cancer Research Campaign Department of Carcinogenesis, Paterson Institute for Cancer Research, Christie Hospital NHS Trust, Manchester, UK.

## Abstract

There is increasing evidence to indicate that O6-methyldeoxyguanosine (O6-MedG) formation in DNA is a critical cytotoxic event following exposure to certain anti-tumour alkylating agents and that the DNA repair protein O6-alkylguanine-DNA alkyltransferase (ATase) can confer resistance to these agents. We recently demonstrated a wide inter-individual variation in the depletion and subsequent regeneration of ATase in human peripheral blood lymphocytes following sequential DTIC (400 mg m-2) and fotemustine (100 mg m-2) treatment, with the nadir ATase activity occurring approximately 4 h after DTIC administration. We have now measured the formation and loss of O6-methyldeoxyguanosine (O6-MedG) in the DNA of peripheral leucocytes of eight patients receiving this treatment regimen. O6-MedG could be detected within 1 h and maximal levels occurred approximately 3-5 h after DTIC administration. Following the first treatment cycle, considerable inter-individual variation was observed in the peak O6-MedG levels, with values ranging from 0.71 to 14.3 mumol of O6-MedG per mol of dG (6.41 +/- 5.53, mean +/- s.d.). Inter- and intra-individual variation in the extent of O6-MedG formation was also seen in patients receiving additional treatment cycles. This may be a consequence of inter-patient differences in the capacity for metabolism of DTIC to release a methylating intermediate and could be one of the determinants of clinical response. Both the pretreatment ATase levels and the extent of ATase depletion were inversely correlated with the amount of O6-MedG formed in leucocyte DNA when expressed either as peak levels (r = -0.59 and -0.75 respectively) or as the area under the concentration-time curve (r = -0.72 and -0.73 respectively). One complete and one partial clinical response were seen, and these occurred in the two patients with the highest O6-MedG levels in the peripheral leucocyte DNA, although the true significance of this observation has yet to be established.


Br. J. Cancer (1994), 69, 853-857 © Macmillan Press Ltd., 1994

### Formation and loss of $O^6$-methyldeoxyguanosine in human leucocyte DNA following sequential DTIC and fotemustine chemotherapy

S.M. Lee[1,2], G.P. Margison[1], N. Thatcher[2], P.J. O'Connor[1] & D.P. Cooper[1]

[1]*Cancer Research Campaign Department of Carcinogenesis, Paterson Institute for Cancer Research and* [2]*Cancer Research Campaign Department of Medical Oncology, Christie Hospital NHS Trust, Manchester, M20 9BX, UK.*

**Summary** There is increasing evidence to indicate that $O^6$-methyldeoxyguanosine ($O^6$-MedG) formation in DNA is a critical cytotoxic event following exposure to certain anti-tumour alkylating agents and that the DNA repair protein $O^6$-alkylguanine-DNA alkyltransferase (ATase) can confer resistance to these agents. We recently demonstrated a wide inter-individual variation in the depletion and subsequent regeneration of ATase in human peripheral blood lymphocytes following sequential DTIC (400 mg m$^{-2}$) and fotemustine (100 mg m$^{-2}$) treatment, with the nadir ATase activity occurring approximately 4 h after DTIC administration. We have now measured the formation and loss of $O^6$-methyldeoxyguanosine ($O^6$-MedG) in the DNA of peripheral leucocytes of eight patients receiving this treatment regimen. $O^6$-MedG could be detected within 1 h and maximal levels occurred approximately 3-5 h after DTIC administration. Following the first treatment cycle, considerable inter-individual variation was observed in the peak $O^6$-MedG levels, with values ranging from 0.71 to 14.3 μmol of $O^6$-MedG per mol of dG (6.41 ± 5.53, mean ± s.d.). Inter- and intra-individual variation in the extent of $O^6$-MedG formation was also seen in patients receiving additional treatment cycles. This may be a consequence of inter-patient differences in the capacity for metabolism of DTIC to release a methylating intermediate and could be one of the determinants of clinical response. Both the pretreatment ATase levels and the extent of ATase depletion were inversely correlated with the amount of $O^6$-MedG formed in leucocyte DNA when expressed either as peak levels ($r = -0.59$ and $-0.75$ respectively) or as the area under the concentration-time curve ($r = -0.72$ and $-0.73$ respectively). One complete and one partial clinical response were seen, and these occurred in the two patients with the highest $O^6$-MedG levels in the peripheral leucocyte DNA, although the true significance of this observation has yet to be established.

Dacarbazine (5-(3,3-dimethyl-1-triazeno)imidazole-4-carboxamide; DTIC) is considered the single most effective chemotherapeutic agent available for the treatment of metastatic melanoma (Comis, 1976; Balch *et al.*, 1989). It undergoes metabolic *N*-demethylation to give the monomethyl triazene, 5-(3-methyl-1-triazeno)imidazole-4-carboxamide (MTIC), which methylates cellular macromolecules including DNA. Among the 12 DNA lesions so formed, $O^6$-methyldeoxyguanosine ($O^6$-MedG) is thought to be the principal cytotoxic product (Meer *et al.*, 1986). It has been shown that resistance to MTIC and related agents involves the activity of the DNA repair protein $O^6$-alkylguanine-DNA alkyltransferase (ATase), which transfers the methyl group from $O^6$-MedG to an internal cysteine residue in an autoinactivating, stoichiometric reaction (Hayward & Parsons, 1984; Gibson *et al.*, 1986; Catapano *et al.*, 1987; D'Incalci *et al.*, 1988; Lunn & Harris, 1988; Foster *et al.*, 1990). The strongest evidence for the cytotoxic effects of $O^6$-alkylguanine on DNA comes from experiments which show that the expression of a transfected prokaryotic or eukaryotic ATase cDNA in mammalian cells protects them against the toxic effects of these agents (Brennand & Margison, 1986; Kataoka *et al.*, 1986; Samson *et al.*, 1986; Jelinek *et al.*, 1988; Kaina *et al.*, 1991).

We recently examined the levels of ATase in human peripheral lymphocytes following combination therapy with DTIC and the chloroethylating agent, fotemustine. ATase activity was depleted and the rate and extent of ATase depletion was patient, dosage and cycle dependent, with the nadir of ATase activity occurring 4-5 h after DTIC administration (Lee *et al.*, 1991, 1993*a*). These findings have been attributed to autoinactivation of ATase during the repair of $O^6$-MedG formed in lymphocyte DNA. It was also shown that fotemustine administration was not associated with ATase depletion in peripheral lymphocytes (Lee *et al.*, 1993*a*).

Correspondence: D.P. Cooper.
Received 15 February 1993; and in revised form 20 December 1993.

The present study investigates the kinetics of formation and loss of $O^6$-MedG in total blood leucocyte DNA to explore possible relationships with changes in ATase levels in peripheral lymphocytes following DTIC administration. These factors may have important therapeutic implications, particularly in combination with the effects of administration of a nitrosourea for which drug resistance can also involve ATase activity (D'Incalci *et al.*, 1988; Pegg, 1990).

### Materials and methods

### *Patients and blood samples*

Details of the individuals studied are shown in Table I. Each patient received DTIC (400 mg m$^{-2}$) by i.v. infusion over 10 min followed 4 h later by fotemustine (100 mg m$^{-2}$) given as a 30 min i.v. infusion. The treatment was repeated every 4 weeks except for patient F.E. (with brain metastasis), who received an additional treatment on day 8. Blood samples (20 ml) were collected just before therapy and at 1, 2, 3, 4, 5, 6 and 18 h after DTIC administration from eight patients in the first treatment cycle. A further five sets of blood samples were collected from four patients who returned for subsequent treatments. Each sample was divided into two universal containers (10 ml each) containing 0.5 ml of 0.5% EDTA, pH 8.0. One half was stored at −20°C prior to DNA extraction and radioimmunoassay for $O^6$-MedG, while the second half was kept at 4°C before isolation of lymphocytes for ATase assays. Approval was given by the local ethical committee and informed consent was obtained from all patients prior to the study.

### *DNA isolation*

Blood samples (10 ml) were thawed, combined with 10 ml of lysis buffer (155 mM ammonium chloride, 10 mM potassium bicarbonate, 2 mM EDTA, pH 7.5) and allowed to stand at 4°C for 30 min. Nuclei were collected by centrifugation at 625 *g* for 10 min at 4°C, resuspended in 1.5 ml of 75 mM sodium chloride, 24 mM EDTA, pH 7.5, and lysed by the

**Table I** Patient characteristics

| *Patient/treatment cycle* | *Age/sex* | *Metastatic sites* | *Response*[a] | *ATase activity* Pretreatment | *ATase activity* Nadir | $O^6$-MedG Peak amount[b] | $O^6$-MedG AUC[c] |
|---|---|---|---|---|---|---|---|
| I.P./1 | 66/F | Nodes/parotid gland | CR | 241 | 137 | 14.3 (h3)[d] | 28.5 |
| I.P./2 | | | | 138 | 90 | 24.7 (h3) | 74.7 |
| I.P./3 | | | | 189 | 77 | 7.6 (h5) | 57.7 |
| K.R./1 | 68/F | Lung/ovaries | PR | 258 | 123 | 12.8 (h3) | 27.3 |
| K.R./2 | | | | 242 | 81 | 1.1 (h5) | 10.9 |
| F.E/1 | 61/M | Lung/brain | NE | 278 | 184 | 7.7 (h4) | 48.2 |
| F.E./2 | | | | 168 | 61 | 8.3 (h2) | 110.3 |
| G.B./1 | 53/M | Skin/nodes/lung | PD | 217 | 72 | 5.7 (h5) | 62.2 |
| G.B./2 | | | | 191 | 118 | 5.6 (h3) | 76.0 |
| J.G./1 | 54/M | Skin/liver | PD | 400 | 196 | 1.0 (h3) | 12.5 |
| M.B./1 | 53/F | Subcutaneous/nodes/bone/ soft tissue | PD | 309 | 163 | 2.0 (h3) | 24.9 |
| M.L./1 | 73/M | Skin/lung/liver/spleen | PD | | | 5.6 (h3) | 68.0 |
| M.H./1 | 40/M | Nodes/soft tissue | PD | 263 | 55 | 0.7 (h3) | 2.0 |

[a]CR, complete response; PR, partial response; PD, progressive disease; NE, not evaluable. [b] Peak amount of $O^6$-MedG in total peripheral leucocyte DNA (μmol mol$^{-1}$ dG). [c]Area under curve of $O^6$-MedG (μmol mol$^{-1}$ dG) *vs* time (hours) from 0 to 18 h except for sample K.R./1, which was integrated from 0 to 4 h. Values calculated following the trapezoid rule using Sigmaplot 5.0 graph-plotting software. [d]Time to reach peak $O^6$-MedG in total peripheral leucocyte DNA (hours).

addition of sodium dodecyl sulphate (SDS) to a final concentration of 1%. The lysate was then incubated overnight at 37°C in the presence of proteinase K (0.1 mg ml$^{-1}$). The following day, 1.5 ml of phenol (saturated with 100 mM Tris-HCl, pH 8.0) was added and after shaking at room temperature for 10 min the mixture was centrifuged at 625 *g* for 5 min. The upper, aqueous phase was re-extracted with phenol as above and residual protein was removed from the final aqueous phase by extraction with 1.5 ml of chloroform-isoamyl alcohol (25:1, v/v). DNA was precipitated by the addition of sodium acetate (10 μl of a saturated solution) and 3.75 ml of ethanol. Following sequential washing in 70% ethanol, ethanol, ethanol-ether (1:1, v/v) and ether, residual solvent was removed by evaporation in a stream of nitrogen. The yield of DNA was 0.2–1.0 mg and the product was free from RNA contamination.

### *$O^6$-alkylguanine–DNA alkyltransferase assay*

ATase activity was measured in cell-free extracts of peripheral blood lymphocytes by monitoring the transfer of radioactive methyl groups from substrate DNA to protein. The substrate was prepared by *in vitro* methylation of calf thymus DNA using nitroso-[$^3$H]methylurea and activity is expressed as fmol of [$^3$H]methyl transferred per mg of protein in the extract (Lee *et al.*, 1991).

### *$O^6$-Methyldeoxyguanosine analysis*

The procedure used for the determination of $O^6$-MedG in DNA has been described in detail elsewhere (Wild *et al.*, 1983; Hall *et al.*, 1991) and is presented here in outline only. DNA was digested enzymatically to nucleosides before ion-exchange chromatography using Aminex A7 resin (BioRad, Hemel Hempstead, UK). The four major deoxynucleosides were separated from $O^6$-MedG by this procedure and were quantified by peak area integration. The putative $O^6$-MedG-containing fractions and control fractions (i.e. similar volumes of buffer from a nucleoside-free region of the column elution profile) were analysed by radioimmunoassay using a monoclonal antibody to $O^6$-MedG (Wild *et al.*, 1983). The results are expressed relative to the amount of dG in the DNA sample. The lower limit of detection using these small amounts of DNA was ~0.4 μmol of $O^6$-MedG per mol of dG.

Where sufficient sample remained, duplicate radioimmunoassay (RIA) determinations were performed, and these indicated an inter-assay variation which was generally $< \pm 20\%$. At values approaching the limit of detection, greater inter-assay variation was apparent, and this approached ± 35% as previously reported (Badawi *et al.*, 1992). Assay variation has also been monitored by inclusion of a control sample which varied from 0.072 to 0.102 pmol of $O^6$-MedG (0.086 ± 0.010, mean ± s.d.) over a period of approximately 3 months.

### Results

### *$O^6$-Alkylguanine–DNA alkyltransferase activity*

The data regarding the changes in ATase activity during combination chemotherapy with DTIC and fotemustine have been reported previously as mean values of a group of patients (Lee *et al.*, 1993*a*). Here, we present the pretreatment and nadir (i.e. the minimum level reached during therapy) lymphocyte ATase activity on an individual basis for all the patients in the present study (Table I). Prior to the first cycle of chemotherapy, ATase activity among the different patients varied by a factor of approximately 2 (range: 217–400 fmol mg$^{-1}$ protein) with a mean value of 289 ± 60 fmol mg$^{-1}$ protein. This value fell to a mean nadir level of 133 ± 54 fmol mg$^{-1}$ protein 4–5 h after treatment and inter-individual variation at this time increased to 3.6-fold (range: 55–196 fmol mg$^{-1}$ protein). Following subsequent cycles of therapy, both the mean pretreatment and mean nadir levels of ATase activity (186 ± 38 and 85 ± 21 respectively) were reduced by a factor of 1.6 when compared with cycle 1.

### *$O^6$-Methyldeoxyguanosine formation in leucocyte DNA*

$O^6$-MedG could be detected in total blood leucocyte DNA shortly after DTIC administration in all patients studied at cycles 1, 2 or 3 of treatment (Figure 1). The kinetics of DNA methylation was broadly similar in all cases: a post-treatment peak in $O^6$-MedG formation occurred at 3–5 h and was followed by a decline in adduct level (Figure 1). However, in some cases (e.g. F.E./1, Figure 1a) there was a tendency for $O^6$-MedG levels to rise again towards the end of the treatment cycle.

There was an approximately 20-fold inter-individual variation in the $O^6$-MedG maxima both among patients in cycle 1 (range: 0.7–14.3 μmol of $O^6$-MedG per mol of dG; 6.4 ± 5.5, mean ± s.d.) and in cycle 2 (range: 1.1–24.7 μmol of $O^6$-MedG per mol of dG; 9.4 ± 8.9, mean ± s.d.). Of the four patients who returned for subsequent courses of DTIC

**Figure 1** $O^6$-MedG levels in peripheral leucocyte DNA in eight patients at various times during cycle 1 (**a** and **b**) or subsequent cycles (**c**) of sequential DTIC ($400\,mg\,m^{-2}$)/fotemustine ($100\,mg\,m^{-2}$) chemotherapy for metastatic melanoma. Arrows indicate the time at which fotemustine was administered.

therapy, two (F.E. and G.B.) achieved peak adduct levels similar to those observed in cycle 1, a third (K.R.) showed an approximately 11-fold reduction and in the fourth patient (I.P.) the peak $O^6$-MedG level in cycles 2 and 3 was increased and decreased, respectively, relative to that observed for cycle 1 (Figure 1 and Table I).

In all but one case (M.H./1), $O^6$-MedG persisted in the DNA for at least 18 h. One individual (F.E.; with brain metastases) returned on day 8 for a second DTIC treatment and $O^6$-MedG was detected in leucocyte DNA at this time at a level of 1.3 $\mu$mol of $O^6$-MedG per mol of dG (Figure 1c). For the remaining cases in which repeat therapy was given at 28 day intervals, residual $O^6$-MedG from previous exposure to DTIC was not detected.

A combined measure of $O^6$-MedG formation and its persistence was obtained for each patient by integration of the area under the $O^6$-MedG concentration–time curve (AUC; Figure 1). The values obtained are shown in Table I. Again, considerable inter-individual variation was apparent with 34-fold and 10-fold differences between the highest and lowest values for cycles 1 and 2 respectively. In general, leucocyte DNA from patients returning for subsequent courses of chemotherapy tended to be more extensively methylated (in terms of the $O^6$-MedG AUC) than in patients receiving the first DTIC treatment (Table I).

*Relationship between lymphocyte ATase activity and leucocyte $O^6$-methyldeoxyguanosine formation in peripheral blood*

The nadir of ATase activity in peripheral lymphocytes occurred 4–5 h after DTIC administration, and this coincided with the peak of DNA methylation in the leucocytes. An inverse correlation was seen between the pretreatment ATase activity and the amount of $O^6$-MedG formed in DNA expressed as either the maxima or the AUC (Figure 2a and c respectively). The extent of lymphocyte ATase depletion (i.e. the difference between pretreatment and nadir ATase activity) was similarly related to the $O^6$-MedG maxima and the AUC (Figure 2b and d respectively). On the other hand, no correlation between the nadir ATase activity level and the extent of $O^6$-MedG formed in DNA was apparent.

### Discussion

DTIC is a prodrug that requires metabolic activation to produce the methylating agent MTIC which can then react with DNA (Meer *et al.*, 1986). The present study demonstrates the presence of $O^6$-MedG in the DNA of peripheral leucocytes from patients receiving combined DTIC/fotemustine therapy and hence the ability of these patients to activate DTIC.

Maximum levels of $O^6$-MedG were observed 3–5 h after DTIC administration, and this coincided with the nadir in ATase activity in peripheral lymphocytes (Lee *et al.*, 1993*a*). Interestingly, DNA single-strand breaks occurring in peripheral blood lymphocytes are also maximal approximately 5 h after DTIC administration (Walles & Ringborg, 1991).

In treatment cycle 1 there was an approximately 20-fold inter-individual variation in the $O^6$-MedG maxima, which was also evident in the group of patients that went on to receive further courses of therapy (Figure 1 and Table I). The $O^6$-MedG AUC gives a combined measure of the formation and persistence of $O^6$-MedG, and these values also show large inter-individual variations, with differences of 34-fold and 10-fold for cycle 1 and subsequent cycles respectively. In some patients, an increase in $O^6$-MedG level was seen at 18 h, and while this effect may be accounted for to some extent by experimental variation (see Materials and methods) reasons for the larger increases seen in patient F.E. (cycles 1 and 2) are unclear. However, similar changes in 7-methyldeoxyguanosine levels have been observed in leucocyte DNA from patients treated with DTIC, and this was attributed to changes in turnover rates of white blood cell subpopulations (van Delft *et al.*, 1992).

Of the four patients returning for additional DTIC therapy, three show an increased $O^6$-MedG AUC with respect to cycle 1. These values (I.P./2, I.P./3, G.B./2 and F.E./2) are associated with relatively low pretreatment ATase levels and form a small cluster in Figure 2c. The effect is most pronounced in F.E., an individual with brain metastases who received a second course of therapy 8 days after the initial treatment when $O^6$-MedG was still detectable in leucocyte DNA (Figure 1c). Presumably, as a consequence of this, ATase activity remained depressed. Such an explanation does not apply to the remaining three instances (I.P./2, I.P./3 and G.B./2) as $O^6$-MedG was not detected at the start of

**Figure 2** The relationship between the peak $O^6$-MedG level (**a** and **b**) or the $O^6$-MedG AUC (**c** and **d**) for leucocyte DNA and pretreatment ATase (**a** and **c**) or ATase depletion (**b** and **d**).

subsequent DTIC cycles, suggesting that in some cases ATase expression does not readily recover.

Clearly, the extent to which these present results can be extrapolated to tumour cells depends on the extent to which $O^6$-MedG levels in leucocyte DNA correlate with those of target tumour DNA. Human tissues and tumours differ greatly in their levels of ATase (D'Incalci *et al.*, 1988), but there are indications that relationships between tissues may exist (Kyrtopoulos *et al.*, 1990): studies in rodent models have shown that DNA methylation occurs to a broadly similar extent in most tissues following administration of methylating agents, even those requiring metabolic activation (Kleihues *et al.*, 1976; Fong *et al.*, 1990), and to a similar extent in leucocyte DNA (Degan *et al.*, 1988).

In fact, in the present study, the kinetics of lymphocyte ATase depletion and leucocyte DNA $O^6$-MedG accumulation does suggest concomitant effects in the two populations of cells. Following DTIC treatment, correlations were seen between the amount of $O^6$-MedG formed in leucocyte DNA (expressed either as the peak value or the AUC) with the pretreatment lymphocyte ATase activity (Figure 2a and c) or with the extent of ATase depletion (Figure 2b and d). Although these relationships are based on a relatively few patients, a similar trend was observed in a study of patients treated with the related drug, procarbazine (Souliotis *et al.*, 1990), and strong correlations between pretreatment ATase activity and both peak $O^6$-MedG levels and $O^6$-MedG AUC have recently been established in patients treated with 1-*p*-carboxyl-3,3-dimethylphenyltriazene (CB10-277; Lee *et al.*, 1993*b*; S.M. Lee *et al.* in preparation). A similar relationship between ATase activity and the amount of $O^6$-MedG in DNA was evident also in analyses of bladder mucosa from individuals exposed to environmental alkylating agents (Badawi *et al.*, 1991). The present results suggest that patients with high initial levels of ATase are therefore able to repair a greater proportion of the $O^6$-MedG resulting from DTIC therapy, whilst the adduct accumulates in the leucocyte DNA of individuals with low pretreatment ATase levels.

Of the eight patients studied, only two (I.P. and K.R.) responded to therapy and, although $O^6$-MedG AUC for both patients was close to the mean value, their leucocyte DNA contained the highest peak levels of $O^6$-MedG after treatment cycle 1 (Table I). This may reflect the need to reach a minimum threshold $O^6$-MedG level before cell killing can occur.

In conclusion, the results reported here lend support to our earlier suggestion that the nadir lymphocyte ATase depletion observed approximately 4 h after 400 mg m$^{-2}$ DTIC is a consequence of DNA methylation, which is also maximal at this time. The wide individual variations in the extent of DNA methylation may result not only from differences in DNA repair capacity but also from differences in the capacity for metabolic activation, uptake and detoxification of DTIC. Use of DNA adduct measurement can therefore provide information on variations in cellular penetration, metabolic activation, distribution and clearance of methylating anti-tumour agents such as DTIC, which in combination with knowledge of ATase activity will permit the design of individualised treatment protocols with improved therapeutic benefit.

We are grateful to Mr J. Davies for technical assistance. This work was supported by the Cancer Research Campaign, the Christie Hospital (NHS) Trust Endowment Fund and the North West Regional Health Authority.

**Abbreviations:** ATase, $O^6$-alkylguanine–DNA alkyltransferase; AUC, area under concentration–time curve; dG, 2′-deoxyguanosine; DTIC (dacarbazine), 5-(3,3-dimethyl-1-triazeno)imidazole-4-carboxamide; MTIC, 5-(3-methyl-1-triazeno)imidazole-4-carboxamide; $O^6$-MedG, $O^6$-methyl-2′-deoxyguanosine.

### References

BALCH, C.M., HOUGHTON, A. & PETERS, L. (1989). Cutaneous melanoma. In *Cancer: Principles and Practice of Oncology*. DeVita, V.T., Hellman, S. & Rosenberg, S.A. (eds), pp. 1499-1542. Lippincott: Philadelphia.

BADAWI, A.F., COOPER, D.P., MOSTAFA, M.H., ABOUL-AZEM, T., MARGISON, G.P. & O'CONNOR, P.J. (1991). $O^6$-alkylguanine-DNA alkyltransferase activity in relation to the promutagenic methylation damage in bladder DNA from humans predisposed to bladder cancer associated with schistosomiasis. *Eur. J. Cancer*, **27** (Suppl. 3), 46.

BADAWI, A.F., MOSTAFA, M.H., ABOUL-AZM, T., HABOUBI, N.Y., O'CONNOR, P.J. & COOPER, D.P. (1992). Promutagenic methylation damage in bladder DNA from patients with bladder cancer associated with schistosomiasis and from normal individuals. *Carcinogenesis*, **13**, 877-881.

BRENNAND, J. & MARGISON, G.P. (1986). Expression in mammalian cells of a truncated *Escherichia coli* gene coding for $O^6$-alkylguanine-DNA alkyltransferase reduces the toxic effects of alkylating agents. *Carcinongenesis*, **7**, 2081-2084.

CATAPANO, C.V., BROGGINI, M., ERBA, E., PONTI, M., MARIANI, L., CITTI, L. & D'INCALCI, M. (1987). *In vitro* and *in vivo* methazolastone-induced DNA damage and repair in L1210 leukemia cells sensitive and resistant to chloroethylnitrosoureas. *Cancer Res.*, **47**, 4884-4889.

COMIS, R.L. (1976). DTIC (NSC-45388) in malignant melanoma: a perspective. *Cancer Treat. Rep.*, **60**, 165-176.

DEGAN, P., MONTESANO, R. & WILD, C.P. (1988). Antibodies against 7-methyldeoxyguanosine: its detection in peripheral blood lymphocyte DNA and potential applications to molecular epidemiology. *Cancer Res.*, **48**, 5065-5070.

D'INCALCI, M., CITTI, L., TAVERNA, P. & CATAPANO, C.V. (1988). Importance of the DNA repair enzyme $O^6$-alkyltransferase (AT) in cancer chemotherapy. *Cancer Treat. Rev.*, **15**, 279-292.

FONG, L.Y.Y., JENSEN, D.E. & MAGEE, P.N. (1990). DNA methyl-adduct dosimetry and $O^6$-alkylguanine-DNA alkyltransferase activity determinations in rat mammary carcinogenesis by procarbazine and *N*-methylnitrosourea. *Carcinogenesis*, **11**, 411-417.

FOSTER, B.J., NEWELL, D.R., LUNN, J.M., JONES, M. & CALVERT, A.H. (1990). Correlation of dacarbazine and CB10-277 activity against human melanoma xenografts with $O^6$-alkyltransferase. *Proc. Am. Assoc. Cancer Res.*, **31**, 401.

GIBSON, N.W., HARTLEY, J.A., LAFRANCE, R.J. & VAUGHAN, K. (1986). Differential cytotoxicity and DNA-damaging effects produced in human cells of the $Mer^+$ and $Mer^-$ phenotypes by a series of 1-aryl-3-alkytriazenes. *Cancer Res.*, **46**, 4999-5003.

HALL, C.N., BADAWI, A.F., O'CONNOR, P.J. & SAFFHILL, R. (1991). The detection of alkylation damage in the DNA of human gastrointestinal tissues. *Br. J. Cancer*, **64**, 59-63.

HAYWARD, I.P. & PARSONS, P.G. (1984). Comparison of virus reactivation, DNA base damage and cell cycle effects in autologous melanoma cells resistant to methylating agents. *Cancer Res.*, **44**, 55-58.

JELINEK, J., KLEIBEL, K., DEXTER, T.M. & MARGISON, G.P. (1988). Transfection of murine multi-potent haemopoietic stem cells with an *E. coli* DNA-alkyltransferase gene confers resistance to the toxic effects of alkylating agents. *Carcinogenesis*, **9**, 81-87.

KAINA, B., FRITZ, G., MITRA, S. & COQUERELLE, T. (1991). Transfection and expression of human $O^6$-methylguanine-DNA-methyltransferase (MGMT) cDNA in Chinese hamster cells: the role of MGMT in protection against the genotoxic effects of alkylating agents. *Carcinogenesis*, **12**, 1857-1867.

KATAOKA, H., HALL, J. & KARRAN, P. (1986). Complementation of sensitivity to alkylating agents in *Escherichia coli* and Chinese hamster cells by expression of a cloned bacterial repair gene. *EMBO J.*, **5**, 3195-3200.

KLEIHUES, P., KOLAR, G.F. & MARGISON, G.P. (1976). Interaction of the carcinogen 3,3-dimethyl-1-phenyltriazene with nucleic acids of various rat tissues and the effect of a protein-free diet. *Cancer Res.*, **36**, 2189-2193.

KYRTOPOULOS, S.A., AMPATZI, P., DAVARIS, P., HARITOPOULOS, N. & GOLEMATIS, B. (1990). Studies in gastric carcinogenesis. IV. $O^6$-Methylguanine and its repair in normal and atrophic biopsy specimens of human gastric mucosa. Correlation of $O^6$-alkylguanine-DNA alkyltransferase activities in gastric mucosa and circulating lymphocytes. *Carcinogenesis*, **11**, 431-436.

LEE, S.M., THATCHER, N. & MARGISON, G.P. (1991). $O^6$-alkylguanine-DNA-alkyltransferase depletion and regeneration in human peripheral lymphocytes following decarbazine and fotemustine. *Cancer Res.*, **51**, 619-623.

LEE, S.M., THATCHER, N., DOUGAL, M. & MARGISON, G.P. (1993*a*). Dosage and cycle effects of dacarbazine (DTIC) and fotemustine on $O^6$-alkylguanine-DNA alkyltransferase in human peripheral lymphocytes. *Br. J. Cancer*, **67**, 216-221.

LEE, S.M., O'CONNOR, P.J., THATCHER, N., CROWTHER, D., MARGISON, G.P. & COOPER, D.P. (1993*b*). Formation and loss of $O^6$-methyldeoxyguanosine ($O^6$-MedG) in peripheral leukocytes of patients receiving dacarbazine or CB10-277. *Proc. Am. Assoc. Cancer Res.*, **34**, 355.

LEE, S.M., O'CONNOR, P.J., THATCHER, N., CROWTHER, D., MARGISON, G.P. & COOPER, D.P. Effects of pretreatment $O^6$-alkylguanine-DNA-alkyltransferase activity on $O^6$-methylguanosine formation in peripheral leukocytes of patients treated with the dimethylphenyltriazene, CB10-277 (manuscript in preparation).

LUNN, J.M. & HARRIS, A.L. (1988). Cytotoxicity of 5-(3-methyl-1-triazeno)imidazole-4-carboxamide (MTIC) on $Mer^+$, $Mer^+$ $Rem^-$ and $Mer^-$ cell lines: differential potentiation by 3-acetamidobenzamide. *Br. J. Cancer*, **57**, 54-58.

MEER, L., JANZER, R.C., KLEIHUES, P. & KOLAR, G.F. (1986). *In vivo* metabolism and reaction with DNA of the cytostatic agent 5-(3,3-dimethyl-1-triazeno)imidazole-4-carboxamide (DTIC). *Biochem. Pharmacol.*, **35**, 3243-3247.

PEGG, A.E. (1990). Mammalian $O^6$-alkylguanine-DNA-alkyltransferase: regulation and importance in response to alkylating carcinogenic and therapeutic agents. *Cancer Res.*, **50**, 6119-6129.

SAMSON, L., DERFLER, B. & WALDSTEIN, E.A. (1986). Suppression of human alkylation-repair defects by *Escherichia coli* DNA-repair genes. *Proc. Natl Acad. Sci. USA*, **83**, 5607-5610.

SOULIOTIS, V.L., KAILA, S., BOUSSIOTIS, V.A., PANGALIS, G.A. & KYRTOPOULOS, A. (1990). Accumulation of $O^6$-methylguanine in human blood leukocyte DNA during exposure to procarbazine and its relationship with dose and repair. *Cancer Res.*, **48**, 2759-2764.

VAN DELFT, J.H.M., VAN DEN ENDE, A.M.C., KEIZER, H.J., OUWERKERK, J. & BAAN, R.A. (1992). Determination of N7-methylguanine in DNA of white blood cells from cancer patients treated with dacarbazine. *Carcinogenesis*, **13**, 1257-1259.

WALLES, S.A.S. & RINGBORG, U. (1991). Induction and time course of DNA single-strand breaks in lymphocytes from patients treated with dacarbazine. *Carcinogenesis*, **12**, 1153-1154.

WILD, C.P., SMART, G., SAFFHILL, R. & BOYLE, J.M. (1983). Radioimmunoassay of $O^6$-methyldeoxyguanosine in DNA of cells alkylated *in vitro* and *in vivo*. *Carcinogenesis*, **12**, 1605-1609.